# Optimization of extraction in supercritical fluids in obtaining *Pouteria lucuma* seed oil by response surface methodology and artificial neuronal network coupled with a genetic algorithm

**DOI:** 10.3389/fchem.2024.1491479

**Published:** 2024-12-10

**Authors:** Alex Chauca-Cerrutti, Marianela Inga, José Luis Pasquel-Reátegui, Indira Betalleluz-Pallardel, Gustavo Puma-Isuiza

**Affiliations:** ^1^ Facultad de Industrias Alimentarias, Universidad Nacional Agraria La Molina, Lima, Peru; ^2^ Grupo de Investigación en Ingeniería y Tecnología Agroindustrial, Facultad de Ingeniería Agroindustrial, Universidad Nacional de San Martín (UNSM), Tarapoto, San Martin, Peru

**Keywords:** circular economy, polyunsaturated fatty acids, polyphenols, antioxidant activity, agro-industrial waste, ecofriendly

## Abstract

When processing lucuma (*Pouteria lucuma*), waste such as shells and seeds is generated, which is a source of bioactive compounds. Recently, lucuma seed (LS), especially its oily fraction, has been studied for containing phytosterols and tocopherols, powerful antioxidants with health benefits. This study proposes lucuma seed oil (LSO) extraction using supercritical fluid (SCF) to improve the quality of the extract and minimize the environmental impact. LS was previously characterized, and the extraction parameters were optimized using a Box-Behnken design, considering temperature (40–60°C), pressure (100–300 bar), and CO_2_ flow rate (3–7 mL/min), applying the response surface methodology (RSM) and neural networks with genetic algorithm (ANN+GA). The optimal parameters were 45°C, 300 bar, and 6 mL/min, obtaining 97.35% of the total oil content. The RSM and ANN+GA models showed R^2^ values of 0.9891 and 0.9999 respectively, indicating that both models exhibited a good fit to the experimental data. However, ANN+GA provided a greater proportion of the total variability, which facilitates the identification of the optimal parameters for the extraction of oil from lucuma seeds. Compared to the Soxhlet method, the LSO obtained by SCF presented better acidity (4.127 mg KOH/g), iodine (100.294 g I_2_/100 g), and refraction indices (1.4710), as well as to a higher content of mono- and polyunsaturated fatty acids. Supercritical CO_2_ extraction is presented as a sustainable green alternative to Soxhlet extraction for extracting oil from lucuma seed due to its high extraction efficiency and similar fatty acid profile.

## 1 Introduction

In the industrial processing of lucuma, waste and byproducts are generated, including seeds (9–11%), peels (11–13%), and rejected fruits from the selection process. Together, these account for nearly 30% of the total net weight (Cari, 2018). This contributes to the generation of waste in Peru, of which more than 50% is made up of plant remains, and approximately only 63.9% is treated. Furthermore, the rest is dumped in illegal landfills, generating environmental pollution and greenhouse gases (Orihuela, 2018). According to Ministerio de Agricultura y Riego (2017), around 5,000 tons of lucuma seed are dumped as part of the agro-industrial waste yearly. Recent studies on reusing these seeds have highlighted their high content of antioxidants, phenolic compounds, and flavonoids (Guerrero-Castillo et al., 2019; Inga et al., 2024). Moreover, its large amount of starch with high amylose concentrations, high viscosity, and excellent water retention capacity makes it a valuable resource for manufacturing polymers (Li et al., 2021). The oil extracted from this seed has also been studied for its application in creams and gels for skin care products due to its healing properties and tissue regenerative capacity (Park et al., 2018), along with its high content of β-sitosterol and antioxidant activity (Guerrero et al., 2021).

Several studies have focused on seeds such as sunflower, sesame, annatto, and citrus, which are known for their biological activities, including antioxidant, antitumor, and antimicrobial properties. These compounds are valuable for treating cardiovascular and neurodegenerative diseases like Alzheimer’s (Ahangari et al., 2021), thus highlighting the importance of efficient oil extraction methods.

The Soxhlet method has traditionally been the standard for oil extraction from seeds. However, Soxhlet extraction relies heavily on organic solvents, which have significant disadvantages. These solvents are often toxic, flammable, expensive, and harmful to the environment, as they can leave chemical residues after extraction (Ahangari et al., 2021).

Novel methods for oil extraction have been applied to the food industry lately that exhibit better performance and avoid hazardous substances production (Lavenburg et al., 2021). Techniques such as microwave-assisted extraction (MAE), ultrasound-assisted extraction (UAE), and especially supercritical CO_2_ extraction (SCF) have emerged as viable alternatives (Danlami et al., 2014).

The application of supercritical fluids (SCF) has been suggested as a method for thermally liable substances extraction that allows obtaining extracts with better characteristics and is a more environmentally friendly alternative (Ye et al., 2019). SCF, such as CO_2_, are used because of their ability to combine the high solvation power of liquids with the high diffusivity and low viscosity of gases, turning them into optimal solvents to extract different compounds (Ahmad et al., 2019). Numerous investigations have applied the SCF technique to extract oils, essential oils, antioxidants, and colorants in different food matrices (De Andrade Lima et al., 2019; Uwineza and Waśkiewicz, 2020; Ahangari et al., 2021). Moreover, Tyśkiewicz et al. (2018) highlight this technology application in extracting bioactive compounds, oils, and phenolics from seeds, peels, and bagasse. In SCF extraction, due to the wide variety of parameters involved in the extraction process, the use of statistical methods such as Response surface methodology (RSM) allows the prediction of the independent variables in a shorter period, fewer experimental runs, and lower cost (Khairul Anwar and Mohamed Afizal, 2015). By applying the mathematical prediction model and experimental designs such as the central composite design (CCD) and Box-Behnken design (BBD), RSM can identify the best result among the surface of possible outputs with statistical reliability and reproducibility (Lamidi et al., 2022). Previous studies have applied the RSM for extraction optimization in food waste. For instance, it has been used in olive-cake oil yield maximization, apple skin essential oil yield optimization, coffee ground oil enrichment with diterpenes, and grape pomace phenolic compounds yield optimization, among others (Barbosa et al., 2014; Ara and Raofie, 2016; Durante et al., 2020; Rodrigues et al., 2023).

Similarly, new optimization methods, such as artificial neural networks, represent a highly active and multidisciplinary area of research because of their ability to solve problems based on large amounts of data that may be distorted, redundant, or nonspecific (Barba Martí and Pallejà Muñoz, 2013). Additionally, artificial neural networks coupled with genetic algorithms (ANNs+GAs) have proven to be an efficient method as they require fewer experimental runs (Nayak et al., 2018; Amdoun et al., 2019) and possess the ability to learn and make predictions without the need to specify a kinetic model’s behavior. Artificial neural networks are now fundamental tools use to tackle a wide range of complex problems in various fields of biological applications (Faiz et al., 2023; Umar et al., 2023; Sabir et al., 2024).

In this context, the extraction of lucuma seed oil (LSO) represents a great research opportunity for the study of its bioactive compounds. Furthermore, supercritical fluid (SCF) technology is a more viable alternative for this purpose due to its advantages over other methods. However, given the complexity of the method and the wide variety of factors implied, it is necessary to apply an optimization method that maximizes performance and preserves the characteristics of LSO. The general objective of the present investigation was to determine the parameters for the yield maximization of LSO extraction using the SCF technique along with RSM and ANN+GA to establish the impact on the oil characteristics compared to another method.

## 2 Materials and methods

### 2.1 Reagent

Folin-Ciocalteau reagent; gallic acid; 2,2-diphenyl-1-picrylhydrazyl (DPPH); 6-hydroxy-2,5,7,8-tetramethylchroman-2,2-Azinobis,3-ethylbenzothiazoline-6-sulphonicacid (ABTS), petroleum ether and carbon tetrachloride were purchased to Sigma-Aldrich (United States), fatty acid identification standards (FAME Mix, C4-C24, 100 mg, Supelco) for fatty acid quantification were purchased from Merck Chemicals Co (Germany). All other reagents were of analytical grade.

### 2.2 Samples

Lucuma seeds of the seda variety were collected from the Topara farm in Chincha, Ica, Peru, during summer, from January to March, as this period corresponds to the peak production due to the fruit’s seasonality. Ten trees were selected within the same field. For each sampling event, fruit units were randomly harvested from the selected trees until 50 kg of fruit was obtained, of which approximately 13% consisted of seeds. This sampling process was repeated in triplicate across three different collection dates to account for potential temporal variations and ensure the representativeness of the samples. All samples followed standardized procedures to maintain integrity throughout the storage and transport phases. This method ensured the homogeneity of the samples while maintaining traceability from the field to the laboratory analysis. Next, seeds were peeled manually and sliced into 2 mm slices using a CL-50 slicer (Robot Coupe), which were dried in an electric oven at 60°C for over 16 h until reaching a moisture content of 5%. The dried slices were ground into powder using a rotor beater mill SR300 (Retsch, German) through a 0.5 mm mesh and sieved to ensure a homogeneous particle size. Samples were weighed and stored at −18°C until use.

### 2.3 Characterization of lucuma seed

LS flour was used to perform the proximal analysis, which consisted of determining the moisture, protein, fat, fiber, and ash content according to the Association of Official Agricultural Chemists (AOAC, 2005) methods.

### 2.4 Determination of the total phenolic content and antioxidant capacity of lucuma seeds

First, the extraction of the bioactive compounds from the lucuma seed was carried out according to the methodology described by Guerrero-Castillo et al. (2019) and Inga et al. (2024), with slight modifications. One gram of sample was mixed with 5 mL of hexane under stirring for 3 h at room temperature (20–25°C). Then, it was centrifuged at 2,700 *g* for 10 min at 4°C, and the supernatant (lipophilic extract) was recovered. The residual pellets was mixed with methanol (80%) in a 1:10 (w/v) ratio under stirring for 3 h. The extract was centrifuged at room temperature at 2,700 g for 15 min. The supernatant (hydrophilic extract) was recovered, and both extracts were stored at −18°C until use.

#### 2.4.1 Total phenolic compounds (TPC)

The methodology described by Singleton and Rossi (1965) was employed for the determination of phenolic compounds, with some modifications. Methanolic extract of LS was diluted with methanol at a 1:10 ratio. 500 μL of the dilution was taken, and 250 μL of the Folin Ciocalteau reagent (1N) was added; the mixture was stirred, and then 1,250 μL of the calcium carbonate solution (7.5%) was added. After incubation for 30 min in dark, absorbance was read in a UV-VIS spectrophotometer (Thermo Fisher Scientific, G10S) at 755 nm. Gallic acid (3.36–4.88 mg/L) was used for the calibration curve. Results were expressed as mg gallic acid equivalents per g of dry weight (GAE/g dw).

#### 2.4.2 Antioxidant capacity using the DPPH method

For the free radical reduction capacity test, the methodology described by Brand-Williams et al. (1995) was used with some modifications. 150 μL of the hydrophilic or lipophilic extracts was pipetted, and 2,850 μL of the DPPH (2,2-diphenyl-1-picrylhydrazyl) solution (0.1107 mmol/L) was added. After incubation for 30 min in dark, absorbance was read in a UV-VIS spectrophotometer (Thermo Fisher Scientific, G10S) at 515 nm. Trolox (150–750 μmol/L) was used for the calibration curve. Results were expressed in μmol Trolox equivalent capacity per gram of dry weight (TEAC/g dw).

#### 2.4.3 Antioxidant capacity using the ABTS method

For measuring antioxidant capacity the methodology described by Re et al. (1999) was followed with some modifications. At first, the ABTS solution was prepared with 7.84 mg/mL of radical ABTS and 1.32 mg/mL of potassium persulfate; the mixture was maintained in the dark at room temperature for 12 h and then diluted with 60 mL of methanol. Hydrophilic or lipophilic extracts were diluted with methanol in a 1:50 ratio. 150 μL of this dilution was pipetted, and 2,850 μL of the ABTS (2,2-Azinobis (3-ethylbenzothiazoline-6-sulphonic acid)) diluted solution was added. After incubation for 60 min in the dark, absorbance was read in a UV-VIS spectrophotometer (Thermo Fisher Scientific, G10S) at 734 nm. Trolox (100–600 μmol/L) was used for the calibration curve. Results were expressed in μmol Trolox equivalent capacity per gram of dry weight (TEAC/g dw).

### 2.5 Experimental design for lucuma seed oil extraction

The selection of the extraction parameters, including temperature, pressure, and flow rate, was based on a combination of previous studies and preliminary experimental trials. Studies that applied supercritical fluid extraction (SCF) to obtain oil from similar matrices were used as foundational references. Specifically, Cordero-Clavijo et al. (2024) used the extraction conditions of 60°C and 400 bar for oil recovery from sacha inchi seeds. Similarly, Cordeiro et al. (2024) investigated oil extraction from piquiá (*Caryocar villosum* (Aubl.) Pers.) at temperature ranges of 40, 50, and 60°C, in combination with pressures of 250, 350, and 450 bar. In addition to these references, preliminary trials were performed to evaluate the effect of extraction time and CO_2_ flow rate on the yield and quality of the oil. These experimental tests and the technical specifications of the extraction system were crucial in fine-tuning the operational parameters of the supercritical extraction (MV-10 ASFE), to ensure optimal conditions for the experimental setup. Therefore, temperature (40, 50 and 60°C), pressure (100, 200, and 300 bar), and CO_2_ flow rate (3, 5, and 7 mL/min) were considered the main factors for the extraction. A randomized Box–Behnken experimental design with 3 factors, 3 levels for each factor, 15 runs in total, and 3 repetitions at the central point was established and codified (Table 1).

**TABLE 1:** Box-Behnken design used in SCF extraction maximization.

Run order	Temperature (°C)	Pressure (bar)	CO_2_ flow rate (mL/min)
1	−1	−1	0
2	1	−1	0
3	−1	1	0
4	1	1	0
5	−1	0	−1
6	1	0	−1
7	−1	0	1
8	1	0	1
9	0	−1	−1
10	0	1	−1
11	0	−1	1
12	0	1	1
13	0	0	0
14	0	0	0
15	0	0	0

### 2.6 LSO extraction

**FIGURE 1:**
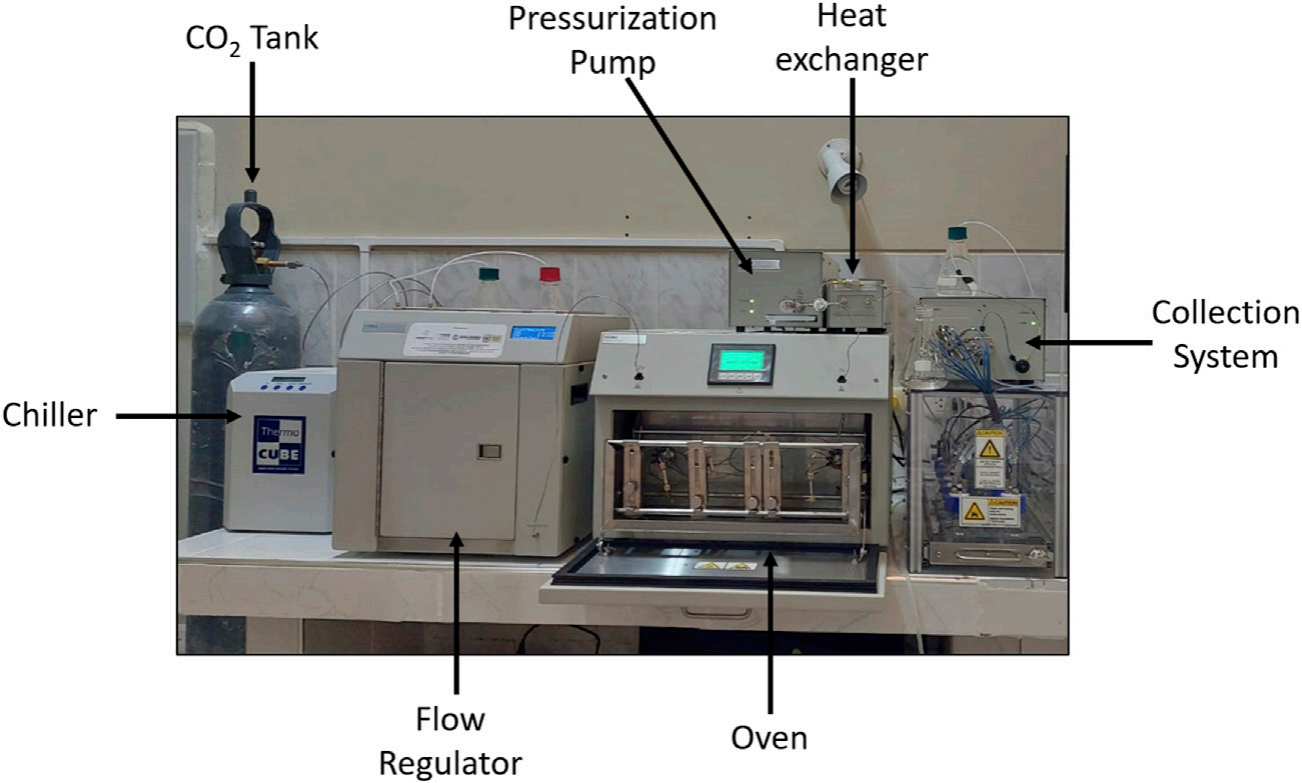
Supercritical fluid extraction equipment.

Several SCF extraction runs were performed over different time ranges to determine the optimal period. The parameters of temperature, pressure, and CO_2_ flow rate used in these runs were according to the central points set in the Box-Behnken design (Botha, 2004; Yang et al., 2011). The extraction kinetics curve established static and dynamic times of 10 and 60 min, respectively. An MV-10 ASFE supercritical extraction equipment (WATERS, USA) was used, which consists of a CO_2_ tank, a chiller, a flow regulator, a pressurization pump, an oven, a heat exchanger, and a collection system (Figure 1). The sample was prepared by weighing 3.0 ± 0.05 g of LS powder and placed in 10 mL cells with spherical soda lime crystals. Cells were placed inside the oven while the solvent injection needle was inserted on one side and the ejector needle on the other. The process began when the outlet valve of the CO_2_ cylinder was opened, and the solvent passed through the chiller to reduce its temperature and ensure that it entered the pump as a liquid. Next, a pump allowed the solvent to be pressurized until supercritical conditions were achieved, while heat was produced by the oven, allowing supercritical temperatures according to the pre-established design. Static extraction began once the solvent reached the desired supercritical conditions, keeping the cell outlet valve closed. After the established time, this valve was opened, and the dynamic extraction phase started, during which the solvent was constantly renewed. The solvent with the solute left the oven, passed through the heat exchanger to reduce its temperature, and then passed through an expansion valve, allowing solvent recovery and solute separation. The extraction yield was calculated by dividing the mass of the extracted oil by the mass of the raw material fed into the system and expressed as a percentage. The oil obtained was stored in amber bottles at freezing temperatures until use.

In addition, LSO extraction was carried out using organic solvents via the Soxhlet method according to the methodology described by Nor and Mohd (2008) with some modifications. Briefly, 15 ± 0.0050 g of the sample was weighed on filter paper and folded to form the cartridge. These were placed in the Soxhlet apparatus, and approximately 200 mL of petroleum ether was added. It was brought to boiling point for about 5 h; later on, the flasks with the oil were placed in an oven at 60°C for 30 min to eliminate the remaining solvent. The yield was estimated in the same way as the extraction by SCF method.

### 2.7 Physicochemical analysis

Oil extracted by both methods was characterized by physicochemical analysis such as acidity index (ISO 660:2020) (International Organization for Standardization, 2020), iodine index (ISO 3961:2018) (International Organization for Standardization, 2018), and refraction index (ISO 6320:2017) (International Organization for Standardization, 2017). The color was also measured using the CIELAB method using a CR-400 colorimeter (Minolta Co. Ltd., Osaka, Japan).

### 2.8 Fatty acid profile

The fatty acid profile was determined according to the method described by Carvalho et al. (2012). Briefly, the oil samples were treated with potassium hydroxide (KOH) in water under continuous stirring for 1 h; then, the fatty acid methyl esters (FAME) were separated by decantation and successive washings. The FAME were passed through a magnesium sulfate filter, and the volatile compounds were removed by vacuum evaporation. The chromatographic analysis was carried out in a gas chromatograph (GC) equipped with an FID (Flame Ionization Detector) (Perkin Elmer Autosystem XL., United States) and a supelcowax-10 silica chromatographic column (0.25 µm, 30 mm × 0.25 mm, SIGMA ALDRICH, Germany). The identification of fatty acids was performed by comparison with the respective standards. Moreover, the percentage concentration of each fatty acid was calculated based on the respective area within the chromatogram.

### 2.9 Response surface methodology (RSM) and statistical analysis

The results obtained from the experimental design were analyzed using the RSM. A quadratic +2 ways model was used in which an analysis of variance (ANOVA) was performed on the regression coefficients with a significance level of α = 0.05. The coefficients with a p < 0.05 were used to develop the reparametrized model, which was analyzed again with an ANOVA at the same confidence interval and significance level. Finally, an adjusted model was obtained that relates the effects of pressure, temperature, and CO_2_ flow rate to the LSO extraction yield. The theoretical results were compared with additional experimental runs to evaluate the adjustment of the model, which allowed the maximization of the LSO extraction yield by SCF method. A Tukey test was used to compare the means of different groups (p < 0.05). Statistical analyses and the maximization model were performed using the STATISTICA V 12 statistical package (StatSoft Inc., United States). Additionally, a desirability index analysis was performed using the model until reaching an index closer to 1.

#### 2.9.1 Neuronal network coupled to a genetic algorithm (ANN+GA)

An Artificial Neural Network (ANN) was built using data generated from the same Box–Behnken design used in the optimization through Response Surface Methodology (RSM) (Puma-Isuiza et al., 2024). In this study, the following values were used for the variables: 40, 50, and 60 for temperature (°C), 100, 200, and 300 for pressure (bar), and 3, 5, and 7 for CO_2_ flow rate (mL/min). These values were combined to obtain 30 values (15 duplicate runs) of the response variable (extraction yield, %). The data was processed using the “neural net” package in R software (Fritsch et al., 2022) for constructing and validating the ANN, of which 80% of the data was used for model construction and 20% for validation. The learning rate was set to 0.01. After building and validating the ANN, optimization was performed using Genetic Algorithms with the “GA” package (Scrucca, 2024). The optimization considered 1,000 iterations, the validated ANN was used as a fitness function, the probability of mutation in a parent chromosome was set to 0.01 and the probability of crossover between pairs of chromosomes was set to 0.8.

## 3 Results and discussions

### 3.1 Characterization of lucuma seed

#### 3.1.1 Proximal analysis

The proximal analysis (Table 2) showed that the crude fat content was 3.21%, which is lower than the 28% and 45% reported for seeds of the same genus, such as *Pouteria sapota*, in the immature and mature states, respectively (Orwa et al., 2009; Solís-Fuentes et al., 2015); as well, lower then the 20.60% reported for seed of *Pouteria campechiana* (Michelle et al., 2021). LS fat content was also lower than those found in seeds commonly used for oil extraction, such as peanuts (46%), safflower (38.50%), chia (30.74%), flaxseed (42.16%) sesame (49.6%), sunflower (49.80%), cottonseed (31.5%), soybean (19.83%) and rapeseed (46.64%) (McKevith, 2005; Lamo and Gómez, 2018). Oilseeds are characterized by having more than 40% crude fat in their composition (Lamo and Gómez, 2018). LS exhibits a relatively lower content of lipids, which may be due to the low presence of oily bodies in the cellular structure (Shang et al., 2020) and the metabolic routes that involve energy that is mainly obtained from the transformation of carbohydrates rather than lipids (Bolaños, 2007; Kelly et al., 2011).

**TABLE 2:** Proximate chemical composition, total phenolic compounds and antioxidant capacity of lucuma seed (dry weight).

Components	Protein	Crude fat	Crude fiber	Ash	Carbohydrates
% Mean	7.51 ± 0.048	3.21 ± 0.006	2.25 ± 0.006	2.51 ± 0.032	84.52 ± 0.011

Phenolic content and Antioxidant Capacity	Hydrophilic	Lipophilic
TPC (μmol GAE/g dw)	31.50 ± 1.14	N.D.
DPPH (μmol TEAC/g dw)	39.24 ± 1.39	N.D.
ABTS (μmol TEAC/g dw)	81.69 ± 7.49	N.D.

Values are presented as means ± standard deviations (n = 3).

(N.D.), not detected.

The carbohydrate content found in LS was 84.52% (Table 2), which is higher than those found in some varieties of the same genus, such as *Pouteria campechiana* (17.62%) (Michelle et al., 2021). This carbohydrate content was also higher than some oilseeds such as cottonseed (21.9%), flaxseed (34.3%), peanuts (12.5%), soybeans (5.1%), sunflower (21.3%), safflower (34.3%) and chia (42.1%) (McKevith, 2005; Ruiz et al., 2013; Lamo and Gómez, 2018); but similar to the carbohydrate content of fruit seeds such as avocado from varieties produced in Africa (74.65%) and Asia (80.12%) (Mahawan et al., 2015; Egbuonu et al., 2018). High levels of carbohydrates are characteristic of non-endospermic seeds such as LS in which these compounds are stored in the cotyledons as the primary source of energy (Aguirre et al., 2018).

It was revealed that LS exhibited a protein content of 7.51% (Table 2), which is lower than the content reported in mature and immature seeds of *P. sapota* (6.47 and 11.34% respectively) and *Pouteria campechiana* (20.73%) (Solís-Fuentes et al., 2015; Michelle et al., 2021). Similarly, the protein concentration of LS is different from those reported in other seeds, such as cotton (32.6%), flaxseed (19.5%), peanuts (25.6%), sesame (18.0%), soybeans (14.0%), sunflower (17.8%), and chia (24.5%) (McKevith, 2005; Ruiz et al., 2013; Lamo and Gómez, 2018), as well as fruits such as mango (5%), avocado (*Persea americana Mill*, 2.64%), and papaya (26.92%) (Noor Raihana et al., 2015; Egbuonu et al., 2018; Kumoro et al., 2020; Bangar et al., 2022). Proteins in seeds are generally made up of enzymes, structural and reserve proteins; these values respond to the needs of each species and tend to increase during the germination period (Bolaños, 2007); therefore, the values observed in different seeds may differ from each other depending on the species and maturity state.

LS fiber content was 2.25% (Table 2), which is lower than the values reported for other seeds of the same, species such as *P. sapota* (25.7%) (Solís-Fuentes et al., 2015); and lower than the values reported in seeds such as cottonseed (5.5%), flaxseed (27.9%), peanuts (6.2%), sesame (7.9%), soybeans (6.1%), sunflower (6.0%), and chia (7.0%) (McKevith, 2005; Ruiz et al., 2013). However, it is similar to the values reported for the seeds of *P. americana* Mill (2.87%) (Egbuonu et al., 2018) and *Mangifera indica L.* (2.38%) (Chaparro et al., 2015). Crude fiber is present in seeds as carbohydrates different from starch, such as lignin, cellulose, and others, as part of cellular structures and tissues. The different proportions of these components depend on factors such as the genetics of the species, the degree of maturity, and production conditions (Badui, 2016; Morais et al., 2017).

Ash content for LS was 2.51% (Table 2); this value is lower than the 3.87% reported in seeds of the same genus as *P. sapota* (Solís-Fuentes et al., 2015). Moreover, this obtained value differs from those found in other seeds such as cottonseed (3.7%), sesame (2.46%), flaxseed (2.14%), sunflower (2.83%), and chia (2.7%) (Ruiz et al., 2013; Lamo and Gómez, 2018). Nevertheless, when comparing the result obtained with the ash content of seeds from fruits such as mango (2.46%) (Chaparro et al., 2015) and avocado (2.40%) (Ifesan et al., 2015), a similarity is noted. Most of the ash content in fruit seeds comprises potassium, calcium, and magnesium as mineral reserves for their development (Bolaños, 2007). The differences observed between these seeds correspond to the requirements of each species since postharvest processes do not influence the ash content (Morais et al., 2017).

These results contribute to understanding the potential of lucuma seed as a source of carbohydrates, protein, crude fiber, crude fat, and ash. This comprehensive approach facilitates exploring diverse applications, thereby both the circular economy and innovation.

#### 3.1.2 Total phenolic compounds (TPC) and antioxidant activity (AC)

LS exhibited a TPC value of 31.50 μmol GAE/g dw (Table 2), which falls within the range reported by Inga et al. (2024) and Guerrero-Castillo et al. (2019), with values of 25.80 μmol GAE/g dw and 52.82 μmol GAE/g dw, respectively. The TPC values are highly influenced by factors such as the ripening stage, cultivation conditions, and postharvest handling; therefore, differences reported for the same seed type may differ considerably (Kosińska et al., 2012). Moreover, the obtained value differs from the TPC for fruits seeds such as avocado (205.2 μmol GAE/g dw), mango (683.7 μmol GAE/g dw), passion fruit (0.004 μmol GAE/g dw) (Soong and Barlow, 2004; Rodriguez-Carpena et al., 2011; Vagula et al., 2017) as well as oilseeds such as white sesame (40.67 μmol GAE/g dw), black sesame (17.24 μmol GAE/g dw), flaxseed (9.40–17.24 μmol GAE/g dw) and soybean (7.60 μmol GAE/g dw) (Alu’datt et al., 2013; Agidew et al., 2021). LS phenolic compounds are mostly organic acid types and flavonoids such as hydroxyundecanoic acid, gallic acid, and gallocatechins (Guerrero-Castillo et al., 2019); due to the wide variety of phenolic compounds that can be found in agro-industrial waste such as seeds. The obtained extracts are affected by factors such as solubility, solute-solvent relationship, temperature, and extraction time, among others, and thus different TPC values can be obtained; which makes their comparison difficult (Rodriguez-Carpena et al., 2011; Alu’datt et al., 2013; Suwal and Marciniak, 2018).

Moreover, the AC value against the DPPH radical for LS was 39.24 μmol TEAC/g (Table 2). This value is closer to the 25.39 μmol TEAC/g reported by Inga et al. (2024), but lower than the 10 mmol TE/g reported for methanolic extracts of other seeds of the same genus such as those of *P. campechiana* (Kong et al., 2013) and higher than the 6.45 μmol TEAC/g found in seeds of *Pouteria caimito* (Vagula et al., 2017). Moreover, it differs from the values reported with the “Hass” and “Fuerte” varieties of *P. americana* Mill (149.73 and 199.26 mmol TEAC/g respectively), amaranth (610 μmol TEAC/g), linseed (5.40 μmol TEAC/g), safflower (9.11 μmol TEAC/g), sunflower (33.96 μmol TEAC/g) (Rodriguez-Carpena et al., 2011; Sreeramulu and Raghunath, 2011; Aguilar-Felices et al., 2019). On the other hand, the value obtained for AA of LS by the ABTS method was 81.69 μmol TEAC/g, similar to the 84.68 μmol TEAC/g reported by Inga et al. (2024); however, it differs from the AC values found in other seeds such as the “Hass” and “Fuerte” varieties of *P. americana* Mill (179.39 and 257.05 mmol TE/g respectively), sunflower (2.21 mmol TEAC/g), amaranth (719 μmol TEAC/g), chia (1.72 mmol TEAC/g) (Rodriguez-Carpena et al., 2011; Sargi et al., 2013; Aguilar-Felices et al., 2019). Variations in values of AC found (both in the ABTS and DPPH radical assays) can be explained by the inherent differences of the food matrices, such as the cultivation conditions and their origin (Serpen et al., 2008). However, the possible existence of non-extractable antioxidants or those linked to insoluble compounds that can generate variation in measurements must be considered (Serpen et al., 2012; Sargi et al., 2013). In food matrices, TPC is directly related to antioxidant activity (Samaniego-Sánchez et al., 2007; Rodriguez-Carpena et al., 2011), which could also explain the reduced values of AC due to the low TPC concentrations found in LS.

### 3.2 LSO extraction optimization model by RSM

The results for the maximization model of LSO using the SCF method are shown in Table 3. The model was reparametrized with factors that showed p < 0.05, thus obtaining a second-grade model that explains the extraction yield as a function of temperature, pressure, and CO_2_ flow rate. No relevant effect was found for the interaction between these factors. However, the coefficients observed in the model revealed a positive sign for the pressure (+1.081863), as well as the for CO_2_ flow rate for its lineal term (+0.173525); meanwhile, temperature exhibited a negative sign for its linear and quadratic terms (−0.119713 and −0.142623 respectively) as well for the quadratic term (−0.139098) of the CO_2_ flow rate. Belhachat et al. (2018) indicate that signs of the coefficients obtained by the regression analysis allow inferring the effect of the factors on the response variable; the ones with a positive sign increase the value on the dependent variable, and those with a negative sign cause it to decrease. Durante et al. (2020) also affirmed that the magnitude of the coefficients indicates the intensity of their effect; therefore, a higher value of these means a more significant relationship with regard of the other coefficients. Therefore, according to the regression analysis shown in Table 3, the independent variable with the most significant relevance in the LSO extraction performance was pressure, followed by CO_2_ flow rate.

**TABLE 3:** Regression analysis for the reparametrized model obtained by the RSM for maximization LSO extraction and the ANOVA result of the model.

Factors	Regression coefficients	p-value
Mean/Interc.	1.860785	—
Temperature (°C) (L)	−0.119713	0.027510
Temperature (°C) (Q)	−0.142623	0.040882
Pressure (bar) (L)	1.081863	0.000351
CO_2_ flow rate (mL/min) (L)	0.173525	0.013380
CO_2_ flow rate (mL/min) (Q)	−0.139098	0.042847
Lack of fit	—	0.196139
R-square	0.9891	
R-adjusted	0.9830	
C.O.V.	6.12%	

Confidence Level (α) = 0.95. Values are presented as the means ± standard deviations (n = 3). L, linear term; Q, Quadratic, term; C.O.V., coefficient of variation.

Table 3 also shows the R-square (0.9891) and R-adjusted values (0.9830) obtained for the LSO extraction maximization model by SCF. According to Peng et al. (2019), R-square values greater than 0.75 ensure that the regression model adequately explains the response variable based on the changes in the independent variables. Hence, in the model obtained for the extraction of LSO, 98.91% of the variation in the responses can be explained by the independent variables, and only 1.09% fails to be explained by it. Tan et al. (2012) also emphasize that the R-adjusted value indicates the precision of the model by eliminating unnecessary factors or variables. If this value is close to the R-square value, it can be deduced that the model has very few non-significant elements. Thus, a high degree of correlation between the expected and actual value can be assured in the model obtained for LSO extraction, in which the R-adjusted value is similar to the R-square.

Additionally, in Table 3, it is shown that pressure has a highly significant effect (p < 0.001), followed by the linear CO_2_ flow rate (p = 0.01338) in LSO extraction. In contrast, the maximization model’s quadratic factors for temperature (p = 0.04088) and solvent flow rate (p = 0.04285) presented less significant effects. On the other hand, Belhachat et al. (2018) state that the lack of fit test measures the level of adaptability of the model to the data obtained from the samples, while the coefficient of variation (COV) is an indicator of dispersion and reproducibility as long as it is less than 10%. In the regression model for maximizing LSO extraction, the lack of fit test produced a non-significant (p > 0.05) result, which demonstrates that the model adequately predicts the performance values based on the independent variables; also, a COV of 6.12% was obtained, which indicates adequate precision and reliability in the reproducibility of the LSO extraction process by SCF.

#### 3.2.1 Parameters effects on LSO extraction

**FIGURE 2:**
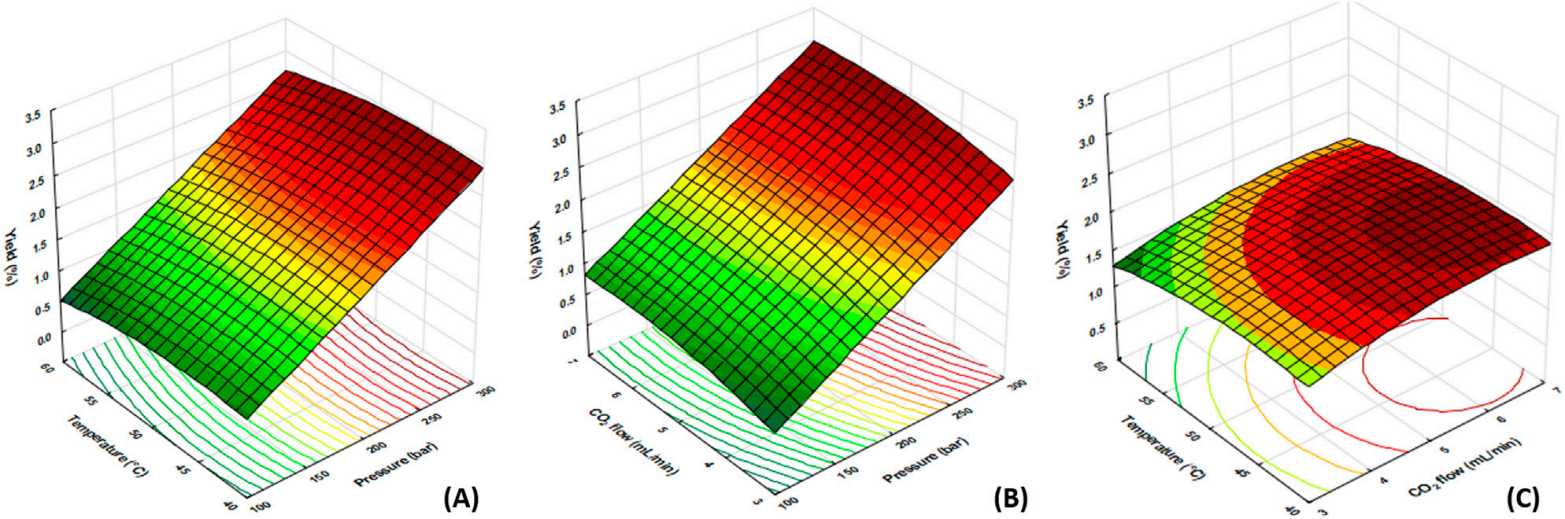
Response surface graph for maximizing yield based on temperature (°C) and pressure (bar) **(A)**; CO_2_ flow rate (mL/min) and pressure (bar) **(B)**; temperature (°C) and CO_2_ flow rate (mL/min) **(C)**.

Figure 2 presents the response surface graphs generated by the RSM for the effects of the different independent variables on the LSO extraction yield by SCF. Figure 2A shows the interaction between temperature and pressure on yield extraction; it is observed that the highest yield is achieved with an increase in pressure greater than 250 bar, while temperature variation (40–60°C) does not generate a significant variation in the response variable. Rebolleda et al. (2012) state that although increases in temperature at a constant pressure achieve higher yields due to an increase in solubility, at pressures greater than 400 bar, the temperature variation negatively impacts the yield. Montañés et al. (2018) explain that temperature can reduce solubility by increasing the vapor pressure of the oil if the pressure crossing point is exceeded, in which there is no longer a synergistic effect between both parameters. This may describe what was observed in the response surface graph since, at a pressure of 300 bar, a higher yield is obtained at 40°C rather than at 60°C. Ahangari et al. (2021) indicate that the increase in pressure is associated with an increase in extraction efficiency as yield increases to a constant point at high pressures (higher than 500 bar), while at low pressures, it increases but decays over time. Muangrat and Pongsirikul (2019) explain that the increase in pressure enhances the extraction of solutes because it increases the density of CO_2_ and, therefore, the solubility of the oil in the solvent. This aligns with what is shown in Figure 2A as the increase in pressures from 100 to 300 bar increases performance for each temperature. Therefore, it can be stated that maximum performance is obtained at pressures greater than 300 bar and a temperature of 40 to 50°C.

Figure 2B shows the interaction between pressure and CO_2_ flow rate on LSO extraction yield. It can be observed that higher yield is achieved with a CO_2_ flow rate higher than 6 mL/min and a pressure higher than 250 bar. Rebolleda et al. (2012) observed that, at a constant solvent flow, the increase in pressure causes an increase in the density of CO_2_ and its solvation power, thus generating a higher extraction yield. This is in agreement with what is observed in Figures 2A, B; for each CO_2_ flow rate level and temperature, an increase in pressure resulted in maximum performance. Molero-Gómez and Martinez de la Ossa (2022) indicated that, at constant pressure, increases in CO_2_ flow rate cause greater performance in the short term, while in prolonged extractions, a lower CO_2_ flow rate maintains a constant solubility in the late phases or when the amount of remaining oil is low. Rai et al. (2017) reported that the increase in the CO_2_ flow rate causes a decrease in the resistance to mass transfer, thereby increasing extraction yield; this is because the number of CO_2_ molecules in the extraction chamber regenerates the contact surface and favors the flow rate of solvent from the inside to the outside. It can be observed in Figure 2B that an increase in extraction performance was generated by the increase in CO_2_ flow rate from 3 to 7 mL/min. Thus it can be deduced from this graph that the maximum yield is obtained at a pressure higher than 250 bar and a flow rate higher than or equal to 6 mL/min.

The response surface graph for the interaction between the independent variables temperature and CO_2_ flow rate on the LSO extraction yield is shown in Figure 2C. It can be seen that, as in Figure 2A, the increases in temperature negatively affects the response variable, and in Figure 2B, yield increases with a higher CO_2_ flow rate. Ahangari et al. (2021) stated that temperature is crucial in SCF extractions. Although increases in this factor promote solubility, it can affect oil quality by promoting oxidation. Gustinelli et al. (2018) indicated that generally, in supercritical CO_2_ extractions, temperatures from 40 to 80°C are used, and variations of 10% may have significant effects on yield extractions. In LSO extraction, these significant variations were observed since it was possible to reach the optimal extraction point at a temperature between 40°C and 50°C. Rai et al. (2017) also indicated that the CO_2_ flow rate commonly used in SCF extractions ranges between 1–10 mL/min. It is known that there is a direct relationship between the CO_2_ flow rate and the extraction yield, although this may vary depending on the nature of the sample. In our study, this trend was evident, obtaining maximum yield values for a CO_2_ flow rate between 6 and 7 mL/min. Although in the response surface graph for the temperature and CO_2_ flow rate variables, it was possible to reach an optimal value (45°C and 6 mL/min approximately).

#### 3.2.2 LSO extraction maximization

**FIGURE 3:**
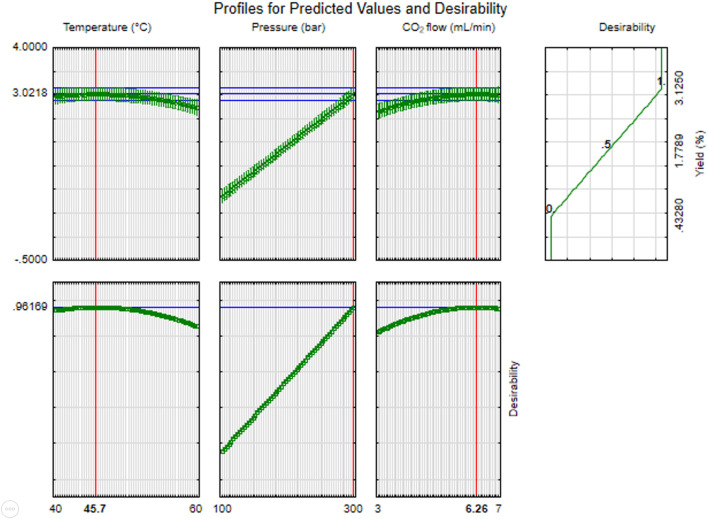
Factors combination for LSO extraction maximization by SCF.

According to the obtained model, the levels of factor combinations in LSO extractions by SCF that achieved the maximum yield were a temperature of 45.7°C, a pressure of 300 bar, and a CO_2_ flow rate of 6.26 mL/min, allowing a yield of 3.0218% with a desirability index of 0.9617 (Figure 3). When close to 1, the desirability index value indicates the most influential parameters for maximizing yield. In order to facilitate the extraction process by the SCF equipment, the maximization values of the parameters were modified to 45°C temperature and 6 mL/min while the pressure remained the same. Three experimental runs were carried out under the previously mentioned conditions, resulting in an average yield of 3.0112 ± 0.041%, similar to the maximum value obtained in the maximization model. The values obtained in the experimental runs according to the maximization parameters and reported by the RSM model were compared through an ANOVA, in which no significant differences were found (p > 0.05). Consequently, the effectiveness of the polynomial model obtained by the RSM for LSO extraction yield maximization could be achieved as the predicted and actual values are quite similar, with an R-square value of 0.9891, RMSE = 0.12, and MAPE = 0.06.

#### 3.2.3 LSO extraction optimization model by ANN+GA

Artificial neural networks are a type of machine learning analysis that has attracted significant attention in the field of prediction and optimization of extraction processes (Abiodun et al., 2018; Song et al., 2024) and the production of unsaturated fatty acids (Izquierdo et al., 2024), because it allows for the modeling of complex non-linear problems (Rebollo-Hernanz et al., 2021).In recent years, the use of genetic algorithms (GA) has been incorporated as they have a high capacity to relate complex and non-linear biological data, even when the exact nature of the relationship between the input parameters and the response variables is not known (Abiodun et al., 2018; Rebollo-Hernanz et al., 2021; Jha and Sit, 2021). In this study, the validated ANN consisted of two layers with five neurons each, obtaining an R^2^ value of 0.9999, root mean squared error (RMSE) = 0.13, and mean absolute percentage error (MAPE) = 0.07. The combination of factors that maximized the extraction yield of lucuma seed oil (3.17%) using ANN+GA was temperature = 50.7°C, pressure = 298.3 bar, and CO_2_ flow rate = 6.22 mL/min.

#### 3.2.4 Comparison of the RMS and ANN+GA prediction models

**FIGURE 4:**
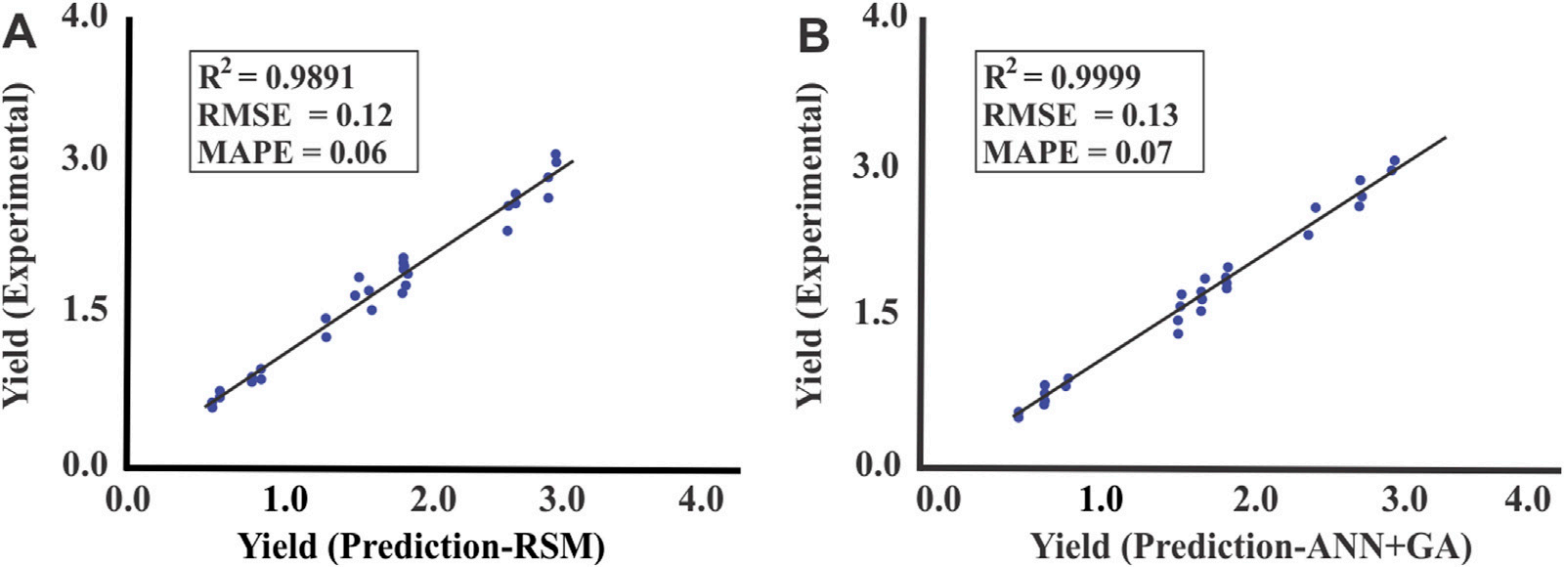
Scatterplot and statistical evaluation of experimental vs. predicted data using RSM **(A)** and ANN+GA **(B)** from the extraction of oil from lucuma seeds.

When correlating the experimental data with the values predicted by the RSM model (Figure 4A) and the ANN+GA model (Figure 4B) for lucuma seed oil extraction, it was observed that the model built by ANN+GA obtained an R^2^ value very close to 1, unlike RSM (R^2^ = 0.9891), which indicates a better fit of the experimental results because the ANN+GA can process data with non-linear functions (Teslić et al., 2019). However, RSM allows the evaluation of the interactions between the levels of the factors under investigation; therefore, the results of both models can be beneficial for the interpretation of the results (Khan et al., 2023). On the other hand, it was observed that both the RSME and MAPE values were similar in both prediction methods, evidencing a good fit. Similar results were reported by various authors for the extraction from bioactive compounds (Teslić et al., 2019; Yang et al., 2019; Khan et al., 2023) and in the oil extraction yield of various seeds (Kostić et al., 2013; Akintunde et al., 2015; Okeleye and Betiku, 2019; Onoji et al., 2019; Esonye et al., 2021).

### 3.3 Physicochemical characteristics and fatty acid content in LSO extracted using the SFC and Soxhlet methods

The yield of LSO extraction obtained using the conventional Soxhlet and SCF methods (Table 4) showed a significant difference (p < 0.05). The SCF method (3.125%) extracted more oil than the Soxhlet method (2.60%). As described in the proximal analysis, the sample had a total oil content of 3.21%, of which the Soxhlet method extracted 83.2%, while the SCF method extracted 97.35% of the total oil content. Similar results have been found in previous studies, such as oil extraction from pumpkin seeds, in which the SCF method managed to recover 100% of the oil while the conventional method only recovered 63.2% (Wenli et al., 2004). Similarly, in the extraction of tea seeds oil, yield values of 31.6% and 30.3% were obtained for extractions with the SCF and conventional methods, respectively (Rajaei et al., 2005). Rombaut et al. (2014) observed that the extraction performance of an oil was mainly linked to its solubility in the solute, where a higher solubility produces a higher yields. This characteristic also depends on the composition of the oil to be extracted. Hence, in the case of LSO, it may be more soluble in supercritical CO_2_ than in organic solvents. Ahangari et al. (2021) mentioned that SCF and Soxhlet extraction yields vary depending on the sample nature and factors such as pressure, temperature, time, flow rate, as well as the use of cosolvents. These can increase or reduce the yield value; therefore, the increment in yield observed in LSO extraction might be explained by the probable higher solubility of the oil in CO_2_ and the difference in temperature and pressure during the process.

**TABLE 4:** Physicochemical characteristics and fatty acid content in LSO extracted by different methods.

Characteristics	Oil extraction method	
	SCF	Soxhlet
Yield (%)	3.125 ± 0.055^a^	2.60 ± 0.12^b^
Total amount of oil (%)	97.35	83.20
Acidity index (mg KOH/g)	4.127 ± 0.052^a^	4.414 ± 0.037^b^
Iodine index (g I_2_/100 g)	100.294 ± 0.188^a^	85.70 ± 0.154^b^
Refractive index	1.4710 ± 0.0002^a^	1.4629 ± 0.0006^b^
Color		
L*	28.14 ± 0.271^a^	25.68 ± 0.118^b^
a*	−3.07 ± 0.066^a^	−2.76 ± 0.129^b^
b*	17.44 ± 0.522^a^	14.06 ± 1.301^b^
Saturated fatty acids (%)	22.11 ± 0.03^a^	27.72 ± 0.44^b^
Monounsaturated fatty acids (%)	32.13 ± 0.14^a^	29.26 ± 0.01^b^
Polyunsaturated fatty acids (%)	45.64 ± 0.11^a^	42.86 ± 0.19^b^
P/S Index	2.06	1.54

Values are presented as the mean ± standard deviation (n = 3). Different letters in each row indicate significant difference (*p* < 0.05).

The acidity index obtained for LSO extracted by supercritical CO_2_ was 4.127 mg KOH/g, which is lower than the 4.414 mg KOH/g obtained for the oil extracted by Soxhlet and lower than the 4.93 mg KOH/g reported for the oil from other seeds of the same genus, such as *Pouteria obovata* (Coca et al., 2017) extracted by the conventional method. Moreover, Wang et al. (2023), when comparing the acidity index of Gardenia fruits oil, they were able to achieve lower values on oils extracted by enzymatic assisted extraction with ultrasonic pretreatment (2.42 mg KOH/g) than oils extracted using the Soxhlet method (3.22 mg KOH/g). A high value of this index indicates a higher amount of free fatty acids present in the oil (Walia et al., 2013) because of the greater degradation of triglycerides by hydrolysis of the ester bonds caused by storage conditions and the extraction method used (Mesquita et al., 2020). Extractions using the Soxhlet method, in which solvents such as hexane or petroleum ether are used, require temperatures higher than 60°C and long periods (Coca et al., 2017). Higher temperatures imply an increase in the vibrations of the molecules, thus favoring bond instability and an increased amount of free fatty acids (Vicentini-Polette et al., 2021).

Previous studies, in which oils obtained by organic solvents and by SCF have been compared, showed that the latter produce oils with a lower acidity index; for instance, Bernardo-Gil and Cardoso-Lopes (2004), obtained 5.5 and 2.3 mg KOH/g for pumpkin seeds oils extracted with n-hexane and with supercritical CO_2_ respectively. Molero-Gómez and Martínez de la Ossa (2002) also managed to reduce the acidity index of borage seed oil from 20.2 to 11.0% through the use of supercritical CO_2_; these authors indicate that the results obtained are due to the temperature (40°C versus 60°C), shorter periods (3 h versus 16 h) and the high selectivity of CO_2_ compared to n-hexane. Furthermore, CODEX (1999) establishes a limit of 0.6 and 4.0 mg KOH/g for refined and unrefined oils, respectively. The values obtained in the LSO by SCF are closer to the established limit than that obtained by organic solvents; however, these are similar to the acidity values reported for crude sunflower oils (4.5 mg KOH/g), coffee bean cake (4.01–6.39 mg KOH/g), and apple seeds (4.28 mg KOH/g) among others (Avramiuc, 2013; Walia et al., 2013; Muangrat and Pongsirikul, 2019).

As shown in Table 4, the iodine index value obtained for LSO by supercritical CO_2_ was 100.294 g I_2_/100 g, which is higher than the 85.70 g I_2_/100 g obtained through extractions using organic solvents and the 70.39 g I_2_/100 g reported for the extract of other seeds from the same genus such as *P. obovata* (Coca et al., 2017). The iodine index indicates the unsaturation quantity and depends on the polyunsaturated fatty acids present in the oils (Walia et al., 2013; Antoniassi et al., 2022). The oil extracted by SCF might have a higher unsaturation content than the oil extracted by organic solvents. Similar results were obtained when comparing SCF and Soxhlet extractions of argan oil, in which 98.5 and 93.4 g I_2_/100 g were obtained, respectively (Taribak et al., 2013). In canola oil, the iodine value increased from 110.5 to 119.5 g I_2_/100 g through SCF (Khattab et al., 2012). Similarly, in rose seed oil, an iodine index of 175 g I_2_/100 g was obtained, which was higher than the 162.5 g I_2_/100 g obtained using the Soxhlet method (Del Valle et al., 2000). These differences are associated with the lower temperature employed, the reduced extraction time, and the capacity of supercritical CO_2_ to extract non-triglyceride lipids, vitamins, and polyphenols (Kattab and Zeitoun, 2013; Triana-Maldonado et al., 2017). However, in previous studies, slight decreases in the iodine index have been observed in SCF extractions at pressures greater than 200 bar and extraction times exceeding 2 h (Muangrat and Pongsirikul, 2019), as well as in extended retention periods and CO_2_ flows rates greater than 0.14 L/min (Triana-Maldonado et al., 2017). Because in the LSO optimal extraction, a pressure of 300 bar was used, the iodine value obtained could have been affected by excess pressure. However, the results obtained are similar to those reported by CODEX (1999) for canola oils (94–120 g I_2_/100 g), cotton (100–123 g I_2_/100 g), corn (103–105 g I_2_/100 g), safflower (80–100 g I_2_/100 g), and sesame (104–120 g I_2_/100 g), among others; therefore, it can be considered an edible oil with regard to its iodine index value.

Furthermore, the refractive index obtained for the LSO extracted with SCF was 1.4710 , which is higher than 1.4629 for that extracted with organic solvents, as shown in Table 4. This characteristic is proportional to the degree of unsaturation and the length of the fatty acid chains present in the oils (Walia et al., 2013). This difference aligns with what was previously observed in the results obtained for the iodine index; that is, the LSO obtained by supercritical CO_2_ has a higher content of unsaturated fatty acids, which are probably long chain such as oleic, linolenic, and linoleic acids, among others (Kostik et al., 2013). Similar results have been observed in previous studies, where Taribak et al. (2013) obtained a slightly higher refractive index in argan oil extracted by supercritical CO_2_ (1.4731) than in that extracted by Soxhlet (1.4719), and Durak et al. (2023) also reported a high refractive index for opoponax oil extracted by SCF (1.4896) compared to that extracted by hydrodistillation (1.4737). However, in optimization studies to obtain sacha inchi oil (Jitpinit et al., 2002), corn germ (Rebolleda et al., 2012), and Jamaica flower seed (Naeem et al., 2019), among others, no significant difference was identified between the oils extracted by supercritical CO_2_, organic solvents, hydro distillation and pressing with regard to refractive index. Therefore, the differences may also be due to the growing conditions, degree of maturity, and seasonality of the fruit (Bolaños, 2007).

Table 4 shows the color results on the CIELAB scale for the LSO extracted by SCF and organic solvents. It can be seen that the oil obtained by supercritical CO_2_ was clearer than the oil extracted by organic solvents, as it has a greater luminosity (28.14 > 25.68), a yellower color (17.44 > 14.06) and a redder color (−2.76 > −3.07). Carotenoids, being responsible for the color of plant oil extracts, are susceptible to oxidation and isomerization reactions because of the action of light, temperature, or the presence of free radicals that produce their degradation and color loss (Amorim-Carrilho et al., 2014). The lower temperatures and shorter time of the supercritical CO_2_ extraction allowed for the better preservation of LSO pigments; hence, its color is brighter than that of the Soxhlet extract. Del Valle et al. (2000) also achieved better color results in rose seed oil extractions by supercritical CO_2_ compared to that extracted by hexane. Similarly, Bernardo-Gil and Cardoso-Lopes (2004) extracted pumpkin seed oil by SCF, which had a more yellowish and discolored hue than that extracted by n-hexane. Bardeau et al. (2015) indicated that, in addition to carotenoids, there were other pigments in oil extractions, such as chlorophylls, because of their lipophilic nature; however, their presence decreases with the maturity of the fruit. In the present study, this would also influence the color difference found in the oil extracts obtained by both methods.

The total fatty acid content for LSO obtained by the conventional Soxhlet and the SCF extractions (Table 4) shows a significant difference (p< 0.05). Also, the existence of a significant difference was observed (p<0.05) between the amount of monounsaturated and polyunsaturated fatty acids in oils extracted by the Soxhlet method (29.26% and 42.86%, respectively) and that by SCF (32.13% and 45.64% respectively). The SCF method provided the highest concentrations of unsaturated fatty acids but a lower amount of saturated acids (27.72% and 22.11%, respectively). This higher concentration of unsaturated fatty acids in the extract obtained by supercritical CO_2_ agrees with the previously observed in the iodine and refraction index results (Table 4), in which the SCF oil presents higher values due to the presence of a higher amount of unsaturation than the oil obtained by organic solvents. Due to its unique solvation properties, SCF selectively extracts bioactive compounds from plant materials, thereby minimizing degradation and preserving the unsaturated fatty acids (Martínez-Ávila et al., 2022). Compared to traditional methods such as Soxhlet, SCF reduces oxidative damage by eliminating solvents, thus lowering the risk of reactions that cause fatty acid deterioration (Lu et al., 2023). These findings are aligned with other studies showing variations in fatty acid profiles based on the extraction conditions (Ramos-Escudero et al., 2019; Madhujith and Sivakanthan, 2019). The results of the polyunsaturated-saturated fatty acid ratio (P/S) (Table 4) show the LSO extracted by SCF had the highest value (2.06) compared to the oil extracted by organic solvents (1.54). This index is the result of the total fatty acid content previously observed and is a determinant of the nutritional value of the oils; a higher index value indicates a significant nutritional relevance (Muangrat and Pongsirikul, 2019). In this context, LSO can be considered an essential fatty acids source for different diets as the obtained P/S index value is similar to those estimated for commonly used oils such as soybean oil (1.43), corn oil (1.87), and grapes seed oil (2.69), among others (Yun and Surh, 2012). However, the higher amount of unsaturated fatty acids found in LSO gives it a more reactive character and, therefore, a shorter shelf life (Bernado-Gil and Cardoso-López, 2004). These characteristics are common in crude oils as they have not undergone a clarification process that can eliminate free fatty acids and other components that promote autooxidation.

#### 3.3.1 Fatty acid profile

**FIGURE 5:**
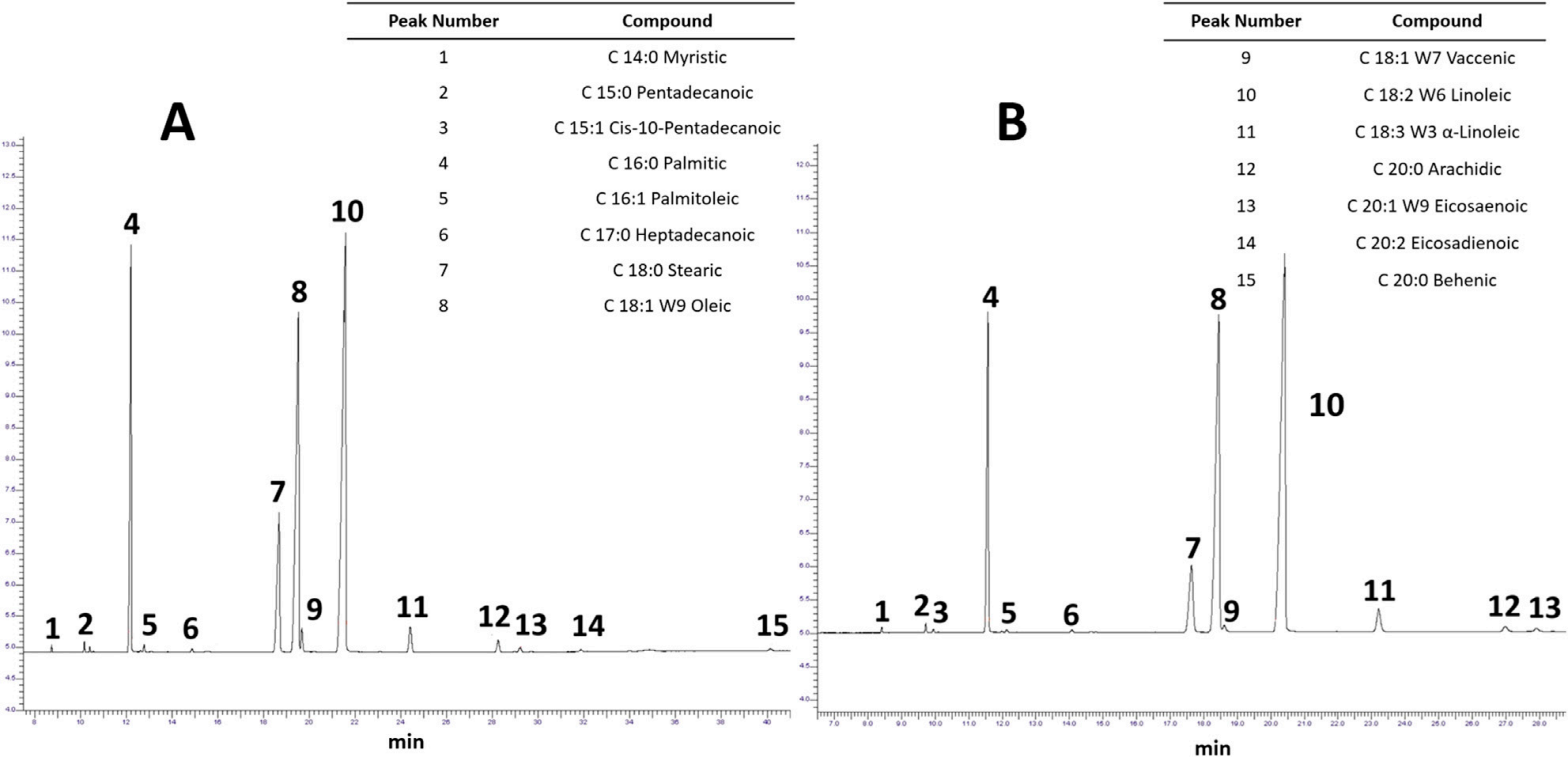
Fatty acid profile for LSO extracted by SCF **(A)** and Soxhlet **(B)**.

The fatty acid profile of LSO extracted by SCF and Soxhlet is shown in Figure 5. A total of 15 peaks were identified in both extracts, with linoleic acid (peak 10), oleic acid (peak 8), palmitic acid (peak 4), and stearic acid (peak 7) being particularly abundant. Vicentini-Polette et al. (2021) obtained sunflower oil by supercritical CO_2_ with a higher content of linoleic acid (64.77%) than the oil extracted by hexane (49.45%) but a lower content of palmitic acid (6.91 vs. 11.62%, respectively) and oleic acid (22.6 vs. 24.83%, respectively). Naeem et al. (2019) also report slightly higher concentrations of linoleic, oleic, and palmitic acid (39.91, 29.38, and 20.52%, respectively) in Jamaican seed oil extracted by SCF compared to oil obtained by organic solvents (36.53, 28.07, and 19.50%, respectively). Khattab and Zeitoun (2013) similarly reported a higher polyunsaturated fatty acid contents in flaxseed oil obtained by supercritical CO_2_ (15.88% linolenic acid and 57.08% α-linolenic acid) than that extracted by the Soxhlet method (15.60 and 56.28%, respectively). Wang et al. (2023) also found a higher content of fatty acids in oils extracted by alternative methods, such as enzymatic assisted extraction (51.02%), than conventional Soxhlet extraction (50.28%). It can be stated that the SCF method allows for the better extraction of fatty acids than the extraction by the Soxhlet method because of the lower temperatures and shorter periods employed. However, Brühl and Matthäus (1999) indicated that this difference was more related to the recovery capacity and selectivity of supercritical CO_2_ than possible hydrolysis during extraction because the latter is verified with diglycerides.

On the other hand, the LSO fatty acid profile is notable for its high concentrations of linoleic (42.23%), oleic (29.52%), and palmitic (15.27%) acids. Similar results were observed in the results reported by Bernardo-Gil and Cardoso-Lopes (2004) for pumpkin seed oil; which exhibited substantial concentrations of linoleic (60%), palmitic (15.2%) and oleic (14.1%) acids; as well as in the fatty acid profiles reported by CODEX (1999) for different oilseeds such as safflower (67.8–83.2% linoleic acid, 8.4–21.3% oleic acid and 5.3–8.0% palmitic acid), sesame (36.9–47.9% linoleic acid, 34.4–45.5% oleic acid and 7.9–12.0% palmitic acid), soybean (48–59% linoleic acid, 17–30% oleic acid and 8–13.5% palmitic acid). The high concentration of these unsaturated fatty acids (linoleic and oleic) makes the LSO an important source of these essential nutrients (Muangrat and Pongsirikul, 2019), along with the beneficial properties that these acids have to reduce blood pressure and serum in the bloodstream (Naeem et al., 2019). Review the Supplementary Material for additional details on the fatty acid result.

## 4 Conclusion

Supercritical CO_2_ extraction is a sustainable green alternative for extracting oil from lucuma seed compared to conventional organic solvent due to its high extraction efficiency (97.35%) under optimal conditions (45.7°C, 300 bar, and a CO_2_ flow rate of 6.26 mL/min). The SCF offers a promising alternative for obtaining LSO with slightly higher extraction yields and a fatty acid profile similar to Soxhlet extraction. The optimization revealed that the ANN+GA presented an R^2^ value close to 1, followed by the RSM. The ANN + GA can process data with non-linear functions, and RSM allows for evaluating the interactions between the levels of the factors being investigated; thus both models are beneficial in optimization processes. The characterization of LSO showed a significant effect of the extraction method on the variable’s acidity index, iodine index, refractive index, and color. The oil extracted by CO_2_ has the best values and highest content of monounsaturated (32.14%) and polyunsaturated (45.64%) fatty acids; as well as, the concentrations of linoleic (42.23%) and oleic (29.52%) acids. These findings highlight the potential applications of lucuma seed oil in the food, cosmetics, and pharmaceutical industries when utilizing supercritical CO_2_ extraction technology.

## Data Availability

The original contributions presented in the study are included in the article/Supplementary Material, further inquiries can be directed to the corresponding author.
