# Supplementary material for: Optimization of extraction in supercritical fluids in obtaining Pouteria lucuma seed oil by response surface methodology and artificial neuronal network coupled with a genetic algorithm
Source: Front Chem. 2024 Dec 10;12:1491479. doi: 10.3389/fchem.2024.1491479 (PMC11666378; doi:10.3389/fchem.2024.1491479)
Supplement: Supplementary file 1 [file Table1.DOCX]

**Supplementary Material**

Tabla A: Fatty Acid Profile of Lucuma Seed Oil

| Fatty Acids | Content (%) | |
| --- | --- | --- |
|  | Soxhlet | SCF |
|  |  |  |
| C 14:0 (Myristic) | 0.16± 0.01^a^ | 0.17± 0.0^a^ |
| C 15:0 (Pentadecanoic) | 0.25± 0.01^a^ | 0.28± 0.0^a^ |
| C 16:0 (Palmitic) | 16.43± 0.16^a^ | 14.10± 0.01^b^ |
| C 16:1 (Palmitoleic) | 0.23± 0.0^a^ | 0.12± 0.01^b^ |
| C 17:0 (Heptadecanoic) | 0.14± 0.01^a^ | 0.12± 0.01^a^ |
| C 18:0 (Stearic) | 9.97± 0.06^a^ | 6.84± 0.03^b^ |
| C 18:1 w-9 (Oleic) | 27.64± 0.01^a^ | 31.40± 0.13^b^ |
| C 18:1 w-7 (Vaccenic) | 1.09± 0.01^a^ | 0.28± 0.01^b^ |
| C 18:2 w-6 (Linoleic) * | 41.04± 0.12^a^ | 43.42± 0.10^b^ |
| C 18:3 w-3 (α-Linolenic) | 1.69± 0.07^a^ | 2.22± 0.01^b^ |
| C 20:0 (Arachidic) | 0.77± 0.01^a^ | 0.61± 0.01^b^ |
| C 20:1 w-9 (Eicosenoic) | 0.30± 0.0^a^ | 0.34± 0.0^a^ |
| C 20:2 (Eicosadienoic) | 0.13± 0.0 | nd |
| C 22:0 (Behenic) | 0.16± 0.0 | nd |

nd: not detectable

|  |  |  |
| --- | --- | --- |

|  |
| --- |
